# Ring finger protein 5 mediates STING degradation through ubiquitinating K135 and K155 in a teleost fish

**DOI:** 10.3389/fimmu.2024.1525376

**Published:** 2024-12-11

**Authors:** Xiaowei Qin, Chuanrui Li, Mincong Liang, Zhen Qian, Yanlin You, Shaoping Weng, Jianguo He, Changjun Guo

**Affiliations:** ^1^ School of Marine Sciences, State Key Laboratory for Biocontrol/Southern Marine Science and Engineering Guangdong Laboratory (Zhuhai), Guangdong Provincial Key Laboratory of Marine Resources and Coastal Engineering & Guangdong Provincial Observation and Research Station for Marine Ranching of the Lingdingyang Bay, Sun Yat-sen University, Guangzhou, China; ^2^ School of Life Sciences, Guangdong Province Key Laboratory for Aquatic Economic Animals, Sun Yat-sen University, Guangzhou, China

**Keywords:** innate immunity, interferons, RNF5, STING, ubiquitination

## Abstract

Stimulator of interferon genes (STING) is a key connector protein in interferon (IFN) signaling, crucial for IFN induction during the activation of antiviral innate immunity. In mammals, ring finger protein 5 (RNF5) functions as an E3 ubiquitin ligase, mediating STING regulation through K150 ubiquitylation to prevent excessive IFN production. However, the mechanisms underlying RNF5’s regulation of STING in teleost fish remain unknown. This study investigated the regulatory role of the mandarin fish (*Siniperca chuatsi*) RNF5 (*sc*RNF5) in the STING-mediated antiviral immune response and identified the specific regulatory sites on *sc*STING. Furthermore, an examination of *sc*RNF5 expression patterns in virus-infected cells revealed its responsiveness to mandarin fish ranavirus (MRV) infection. The ectopic expression of *sc*RNF5 suppressed *sc*STING-mediated IFN signaling and facilitated MRV replication. Co-immunoprecipitation experiments indicated an interaction between *sc*RNF5 and *sc*STING. The further experiments demonstrated that *sc*RNF5 exerted its inhibitory effect by promoting the degradation of *sc*STING, which was observed to be blocked by MG132 treatment. Ubiquitination assays with various *sc*STING mutants showed that *sc*RNF5 catalyzed the ubiquitination of *sc*STING at K135 and K155 residues. Furthermore, we provided evidence that *sc*RNF5 significantly attenuated *sc*STING-dependent antiviral immunity by targeting negative regulators within the *sc*STING signaling cascade. This study underscored that RNF5 negatively regulated the STING-mediated IFN signaling pathway in mandarin fish, attenuated STING’s antiviral activity, and facilitated STING degradation via the ubiquitin-proteasome pathway at two novel lysine sites (K135 and K155). Our work offered valuable insights into the regulatory mechanisms of STING-mediated signaling in teleost fish, paving the way for further research.

## 1 Introduction

Stimulator of interferon genes (STING), also known as TMEM173, MITA, ERIS, MPYS or NET23, is a transmembrane adaptor protein critical for the innate immune response to pathogenic cytoplasmic DNA (1, 2). Upon recognition of pathogenic DNA, signals converge on STING. The activated STING is then translocated to perinuclear endosomes along with TANK-binding kinase 1 (TBK1) (3). TBK1 phosphorylates and activates interferon regulatory factors (IRFs) and nuclear factor kappa B (NF-κB), thus initiating the production of type I interferon (IFN-I) and other immune response genes (1, 4, 5). STING activity is tightly regulated through various post-translational modifications, including phosphorylation and ubiquitination in mammals (6). Among these regulatory mechanisms, ubiquitination of STING has emerged as a key process, exerting both positive and negative effects on its antiviral signaling (7).

Ring finger protein 5 (RNF5), also referred to as G16 or RMA1, is an E3 ubiquitin ligase localized to the endoplasmic reticulum (8). Early research indicates that RNF5, identified as a novel regulator of cell movement, suppresses cell motility by promoting paxillin ubiquitination (9). Subsequent studies have demonstrated the RNF5’s involvement in muscular dystrophies, myogenesis, and the regulation of various malignancies (9–11). Furthermore, RNF5 facilitates K48-linked ubiquitination and degradation of STING and mitochondrial antiviral signaling protein (MAVS), thereby suppressing host antiviral responses (12, 13). RNF5 inhibits STING/IRF3 signaling, limiting the antiviral response to type I IFN in herpes simplex virus keratitis (14). The V protein of Newcastle disease virus interacts with MAVS, promoting K48-linked ubiquitination and RNF5-mediated degradation (15). In zebrafish, the NLRX1 isoform specifically recruits the E3 ubiquitin ligase RNF5, facilitating K48-linked ubiquitination and leading to proteasome-dependent degradation of STING, ultimately downregulating the IFN antiviral response (16). Beyond degrading STING and MAVS, RNF5 also directly inhibits the function of other virus-associated proteins, potentially modulating the viral life cycle. For instance, RNF5 targets viral antiviral signaling proteins for degradation, thereby hindering viral proliferation and spread (17). Moreover, studies have shown that RNF5 competitively inhibits viral proteins, impeding the replication of specific viruses, further supporting its role in the antiviral process (18). STING homologs have been identified in various teleost fish species, such as grass carp (*Ctenopharyngodon idella*), grouper (*Epinephelus coioides*), black carp (*Mylopharyngodon piceus*) and mandarin fish (*Siniperca chuatsi*), playing a crucial role in the antiviral signaling pathway (19–25). In black carp, RNF5 mediates STING and MAVS ubiquitination, thereby negatively regulating the antiviral innate immune response (26, 27). However, the precise mechanism by which RNF5 regulates STING activity via ubiquitination and the specific site of STING ubiquitination, remains unknown in teleost fish.

Mandarin fish (*S. chuatsi*) is a highly valuable aquaculture species in China’s aquaculture market (28). Intensive aquaculture practices, driven by the human demand for animal protein, have led to high-density farming of mandarin fish. However, these practices frequently result in disease outbreaks, with the mandarin fish ranavirus (MRV), a double-stranded DNA virus belonging to the *Ranavirus* genus of *Iridoviridae* family, posing a significant threat (29). Therefore, elucidating the innate immune regulatory mechanisms in fish will advance research on antiviral immunity and therapeutics.

In this study, the regulatory role of the *S. chuatsi* RNF5 (*sc*RNF5) in the STING-mediated antiviral immune response and pinpointed its specific regulatory sites were investigated. Our findings will offer valuable insights for the further elucidating the regulatory mechanisms underlying STING-mediated signaling in teleost fish.

## 2 Materials and methods

### 2.1 Animal, cells, and virus

Mandarin fish (*S. chuatsi*), averaging 50 grams in weight, were sourced from a farm in Guangdong province, China. Prior to the experiment, the fish were acclimated for two weeks in a laboratory-controlled, recirculating freshwater system at 27°C. All animal procedures were conducted in accordance with Guangdong Province’s animal experimentation regulations and approved by the ethics committee of Sun Yat-sen University. The mandarin fish fry (MFF-1) cell line was cultured in Dulbecco’s Modified Eagle Medium (DMEM; Gibco, Grand Island, USA), supplemented with 10% fetal bovine serum (FBS; Gibco, Grand Island, USA), and maintained at 27°C in a humidified atmosphere with 5% CO_2_ (30). Fathead minnow (FHM) cells (ATCC CCL-42) were maintained in M199 medium supplemented with 10% FBS (Gibco, Grand Island, USA) at 27°C (31). The mandarin ranavirus (MRV) strain NH-1609 (Accession number: MG941005) was isolated from moribund hybrid mandarin fish in Naihai, Guangdong, China, and is preserved in our laboratory (31).

### 2.2 Antibodies and reagents

Mouse monoclonal anti-c-Myc antibody and mouse monoclonal anti-Flag antibody were obtained from Sigma–Aldrich (St. Louis, USA). The anti-Ha tag antibody was purchased from Cell Signaling Technology (CST, Danvers, USA). Anti-GAPDH was obtained from Abways (Shanghai, China), and rabbit polyclonal anti-mrvORF097L antibody was produced in our laboratory (32). Secondary antibodies included horseradish peroxidase (HRP)-conjugated goat anti-mouse IgG (H+L) and HRP-conjugated goat anti-rabbit IgG (H+L), both supplied by Promega (Madison, USA). The proteasome inhibitor MG132, cycloheximide (CHX), 3-Methyladenine (3-MA), and chloroquine (CQ) were all purchased from MedChemExpress (New Jersey, USA). Lodoacetamide (IAA) was obtained from CST (Danvers, USA).

### 2.3 Protein analysis

For protein domain analysis, SMART (Simple Modular Architecture Research Tool, a web resource for the identification and annotation of protein domains and the analysis of protein domain architectures was used to compartmentalize the protein domains of *sc*RNF5 in Genomic Mode (33). For evolutionary tree, protein sequences were retrieved from GenBank (34) and RefSeq (35) databases and aligned by ClustalW in MEGA v11.0.13 (a software contains a large collection of methods and tools of computational molecular evolution) (36). Then the evolutionary tree was constructed in MEGA using the Neighbor-Joining method with 1,000 bootstrap replications, Jones-Taylor-Thornton (JTT) model, and complete deletion. The evolutionary tree was visualized using iTOL (an online tool for the display, annotation and management of phylogenetic and other trees) (37), incorporating cartoon elements from the Generic Diagramming Platform (a comprehensive database of high-quality biomedical graphics) (38). For protein three-dimensional (3–D) structure predictions, protein sequences were obtained from RefSeq databases, and Alphafold Server (a web server can predict the joint structure of complexes including proteins, nucleic acids, small molecules, ions and modified residues) (39) was employed to predict the protein structures. For protein amino acid sequence alignment, protein sequences were obtained from GenBank and RefSeq databases and DNAMAN v9.0 (a one-for-all software package for molecular biology applications) was used to align with Dynamic Alignment and Default Parameters.

### 2.4 Plasmid construction and cell transfection

Total RNAs were extracted, and cDNAs were synthesized following previously described methods (25). *sc*RNF5 and *sc*STING were constructed via polymerase chain reaction (PCR), cloned, and then inserted into pCMV-Myc-N and pCMV-Flag-N vectors (Takara, Japan). This process generated the plasmids: pCMV-Myc-*sc*RNF5, pCMV-Flag-*sc*STING, pCMV-Flag-*sc*STING (1–140), pCMV-Flag-*sc*STING(141-417), pCMV-Flag-*sc*STING(1-180), and pCMV-Flag-*sc*STING(181-417) plasmids Various STING constructs, including Ha-*sc*STING(1-180)KR, Flag-*sc*STINGCKR, Flag-*sc*STINGCKRK20, Flag-*sc*STINGCKRK95, Flag-*sc*STINGCKRK117, Flag-*sc*STINGCKRK122, Flag-*sc*STINGCKRK135 and Flag-*sc*STINGCKRK155, were synthesized by Beijing Tsingke Biotechnology (China). The primers used for gene cloning, which contained restriction enzyme sites, are listed in **Supplementary Material 1**. The plasmids were transfected into FHM cells using Fugene HD (Promega, USA), and into MFF-1 cells using Transfect EZ 3000 Plus (eLGbio, China), adhering to the manufacturers’ standard protocols.

### 2.5 Tissue expression profiles of *sc*RNF5

To examine the tissue distribution of *sc*RNF5 in healthy fish, total RNAs were extracted from various tissues including the gills, fins, spleen, intestine, brain, head kidney, hind kidney, middle kidney, blood, fat, heart, liver, and muscle. Extraction was performed using the SV Total RNA Isolation Kit (Promega, USA) following the manufacturer’s instructions. The expression levels of *sc*RNF5 in these tissues were then quantified by quantitative reverse-transcription PCR (qRT-PCR) using the primers listed in **Supplementary Material 1**.

### 2.6 Dual-luciferase reporter assays

MFF-1 cells were cultured in 24-well plates and co-transfected with pRL-TK and IFN-β-Luc plasmids (Promega, USA), and the indicated plasmids or empty vector. After 36 h, cells were harvested, lysed in 200 µL of Passive Lysis Buffer (Promega, USA), and subjected to the Dual-Luciferase Reporter Gene Assay Kit (Promega, USA) following the manufacturer’s instructions. Luciferase activities were measured using Glomax instrument (Promega, USA). Experiments were performed in triplicate, and data represented the mean of at least three independent experiments. All experiments were conducted in at least three independent trials with three technical replicates.

### 2.7 RNA extraction and qRT-PCR analysis

Total RNA was extracted from cells or tissues using the SV Total RNA Isolation Kit (Promega, USA) and reverse transcribed to synthesize first-strand cDNA using the Evo M-MLV qPCR RT Kit (AG, China), following manufacturers’ protocols. qRT-PCR reactions were performed with SYBR premix ExTaq (Takara, Japan) on a LightCycler 480 instrument (Roche Diagnostics, Switzerland), as previously described (40). Expression levels of immune-related genes and viral genes were normalized to *scβ-actin* gene. Primer sets for the *scRNF5, scSTING, scβ-actin, mrvMCP, mrvICP18*, and *mrvDNA polymerase* (*mrvDNA-Poly*) genes are described in **Supplementary Material 1**. Data were analyzed using Q-gene statistics add-in and unpaired sample *t*-test. Statistical significance was set at *p*<0.05, with high significance at *p*<0.01. All data were expressed as mean ± standard deviation (SD).

### 2.8 Co-immunoprecipitation and western blot analysis

For Co-IP, FHM cells were seeded in 25 cm² dishes, cultured overnight, and co-transfected with 4 μg of vector. After 24 h, cells were lysed using IP lysis buffer (Beyotime, China) with protease inhibitor cocktail III (Merck Millipore, USA), and whole-cell lysates were centrifuged at 12,000 ×g for 10 minutes at 4°C. Supernatants were collected for immunoprecipitation (IP) using anti-Flag or anti-Myc beads (Alpa Life Bio, China), as previously described [36]. Beads were added to the supernatant, incubated for 4 h at 4°C, washed, and proteins were collected by centrifugation and heated to 100°C for 10 minutes for WB analysis. Protein bands were detected using the High-sig ECL western blotting substrate (Tanon, China) and visualized with the Amersham Image Quant 800 system (Cytiva, USA).

### 2.9 Viral titer determination

MFF-1 cells were seeded into 96-well plates, cultured overnight to exceeding 80% confluence, and infected with serial 10-fold dilutions of MRV-containing samples, with eight wells per dilution. Eight wells containing 100 μL of virus-free culture medium served as negative controls, and plates were incubated at 27°C for seven days, as previously described (41, 42). The number of positive and negative wells was recorded to calculate the TCID_50_ using the Spearman-Karber method, as previously described (41, 42).

## 3 Results

### 3.1 Sequence and phylogenetic analysis of *sc*RNF5

**Figure 1:**
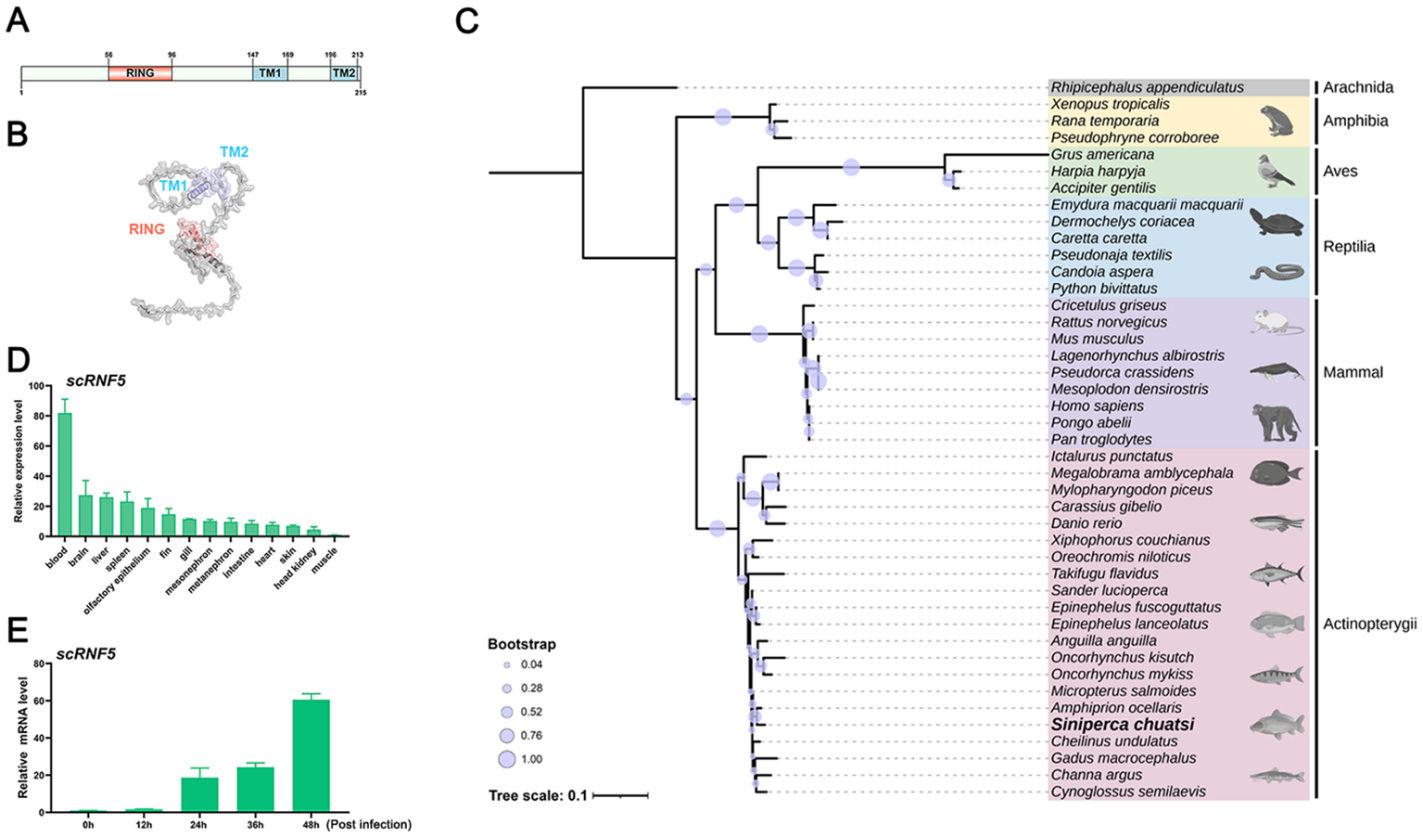
Molecular characteristics of *sc*RNF5. **(A, B)** Domain and 3–D structure prediction of *sc*RNF5. **(C)** A phylogenetic tree was generated using the MEGA v11.0.13 program, based on RNF5 sequences from various vertebrate species (GenBank accession numbers are provided in the **Supplementary Material 2**). **(D)** Tissue distributions of *sc*RNF5 expressions in different tissues of mandarin fish. Samples were collected from the blood, brain, liver, spleen, fin, gill, intestine, heart, skin, head kidney, and muscle of three healthy mandarin fish, and the expression levels of the *sc*RNF5 were detected by qRT-PCR. **(E)** Expression of *sc*RNF5 in response to MRV infection. *sc*RNF5 mRNA levels in MFF-1 cells were measured by qRT-PCR and normalized to the reference *β-actin* gene after infection with MRV at 0.1 MOI.

To investigate the role of RNF5 in mandarin fish, we cloned the full-length cDNA of *sc*RNF5 gene and constructed expression plasmids. Sequence analysis showed that the *sc*RNF5 coding sequence (NCBI accession number: XP_044026571.1) consists of 648 nucleotides. It encodes a 216 amino acids (aas) protein with two transmembrane domains (TM) and a ring-finger domain (RING), weighting 23.2 kDa (**Figure 1A**). 3–D structure predictions showed that the spatial structure of *sc*RNF5 and the spatial arrangement of its domains were similar to its mammalian ortholog (**Figure 1B**). Phylogenetic analysis clustered *sc*RNF5 with RNF5 proteins from other fish, distinguishing it from the other RNF5 family members (**Figure 1C**; **Supplementary Material 2**).

### 3.2 Expression patterns of *sc*RNF5 in healthy fish and virus-infected cells

To assess the tissue-specific expression of RNF5 in mandarin fish, we used qRT-PCR to analyze transcript levels of *sc*RNF5 across various tissues, including blood, brain, liver, spleen, fin, gill, intestine, heart, skin, head kidney, and muscle. As shown in **Figure 1D**, *sc*RNF5 transcripts were detected in all sampled tissues from healthy individuals, with notably higher expression in blood, approximately 81-fold higher than in muscle, highlighting its tissue-specific expression. In MRV-infected cells, *sc*RNF5 expression exhibited a time-dependent increase, with up-regulation levels reaching 18-, 24-, and 60-fold at 24, 36, and 48 h post-infection, respectively (**Figure 1E**), suggested that *sc*RNF5 responded to MRV infection.

### 3.3 *sc*RNF5 facilitated MRV replication

**Figure 2:**
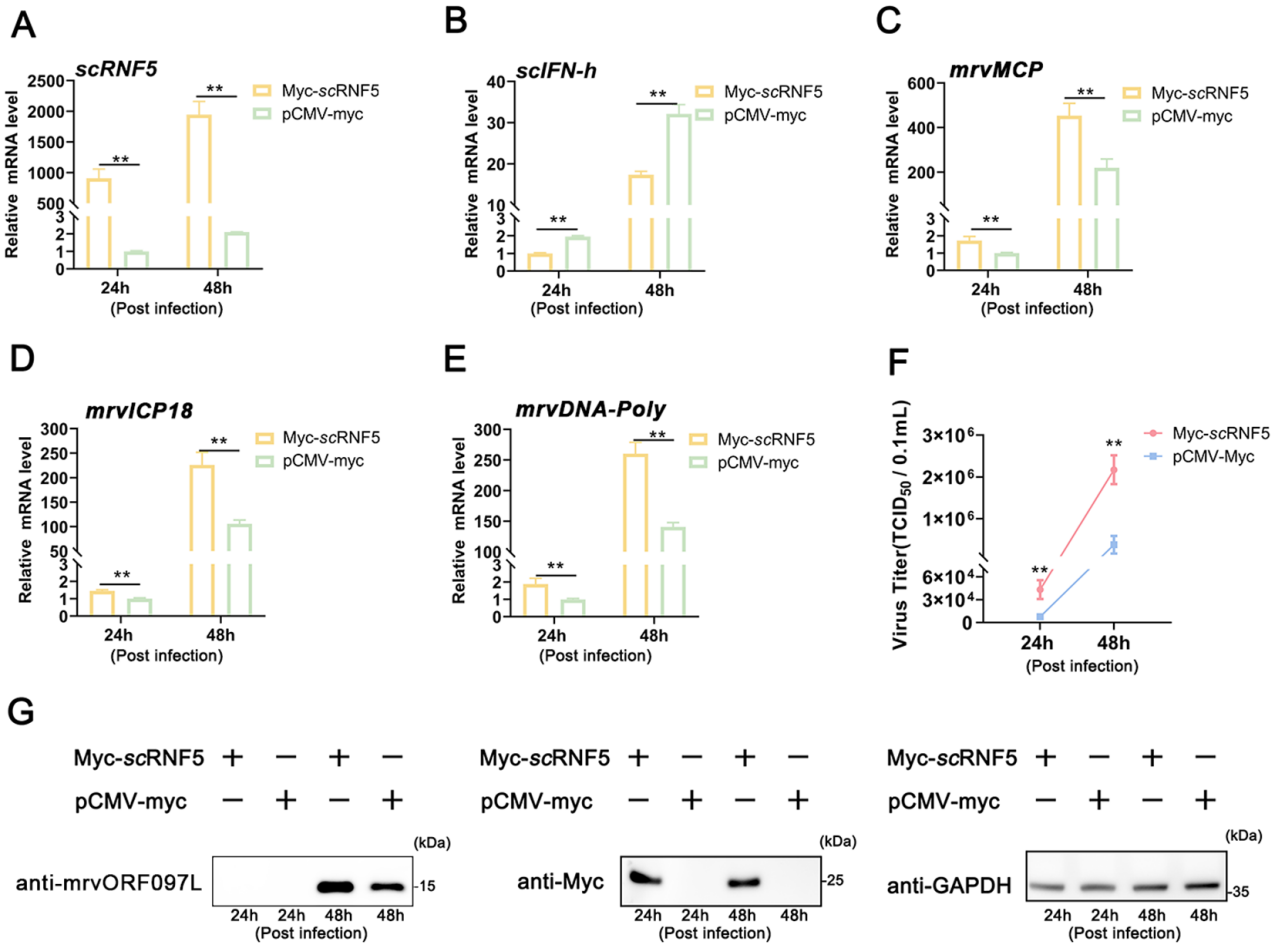
Overexpression of *sc*RNF5 enhances MRV infection. Cells were transfected with *sc*RNF5-myc or pCMV-myc for 24 h, then infected with MRV, and harvested at the indicated time points for qRT-PCR analysis. **(A)** Expression levels of *sc*RNF5 in MRV-infected cells at indicated times. **(B)** Expression levels of *scIFN-h* genes in MRV-infected cells at various time points. **(C–E)** Expression levels of *mrvMCP*, *mrvICP18*, *mrvDNA-Poly* genes in MRV-infected cells at indicated times. For qRT-PCR analysis, the baseline was established as the group exhibiting the lowest relative expression among all groups, with this value set to 1. All groups represented different time points following viral infection. **(F)** Virus infection was measured on a 96-well cell culture plate via the finite dilution method. **(G)** Protein levels of mrvORF097L were detected via WB analysis. Vertical bars represent ± SD (*n* = 3). Statistical significance is indicated by asterisks, with ** referring to *p* < 0.01.

MFF-1 cells expressing *sc*RNF5 or a control vector (pCMV-myc) were infected with MRV, and subsequent cells were then harvested for qRT-PCR, WB, and TCID_50_ analysis. The results showed that overexpression of *sc*RNF5 (**Figure 2A**) reduced the expression level of *scIFN-h* gene (**Figure 2B**); but the expression levels of the and MRV viral genes (*mrvMCP, mrvICP18, mrvDNA-Poly*) were significantly higher in cells expressing *sc*RNF5 compared to the control group (**Figures 2C–E**). Furthermore, both the levels of viral titers (**Figure 2F**) and viral protein mrvORF097L (**Figure 2G**) were markedly elevated in the *sc*RNF5 group compared to the control. These results indicated that *sc*RNF5 facilitated MRV replication.

### 3.4 *sc*RNF5 suppressed *sc*STING-mediated IFN signaling

**Figure 3:**
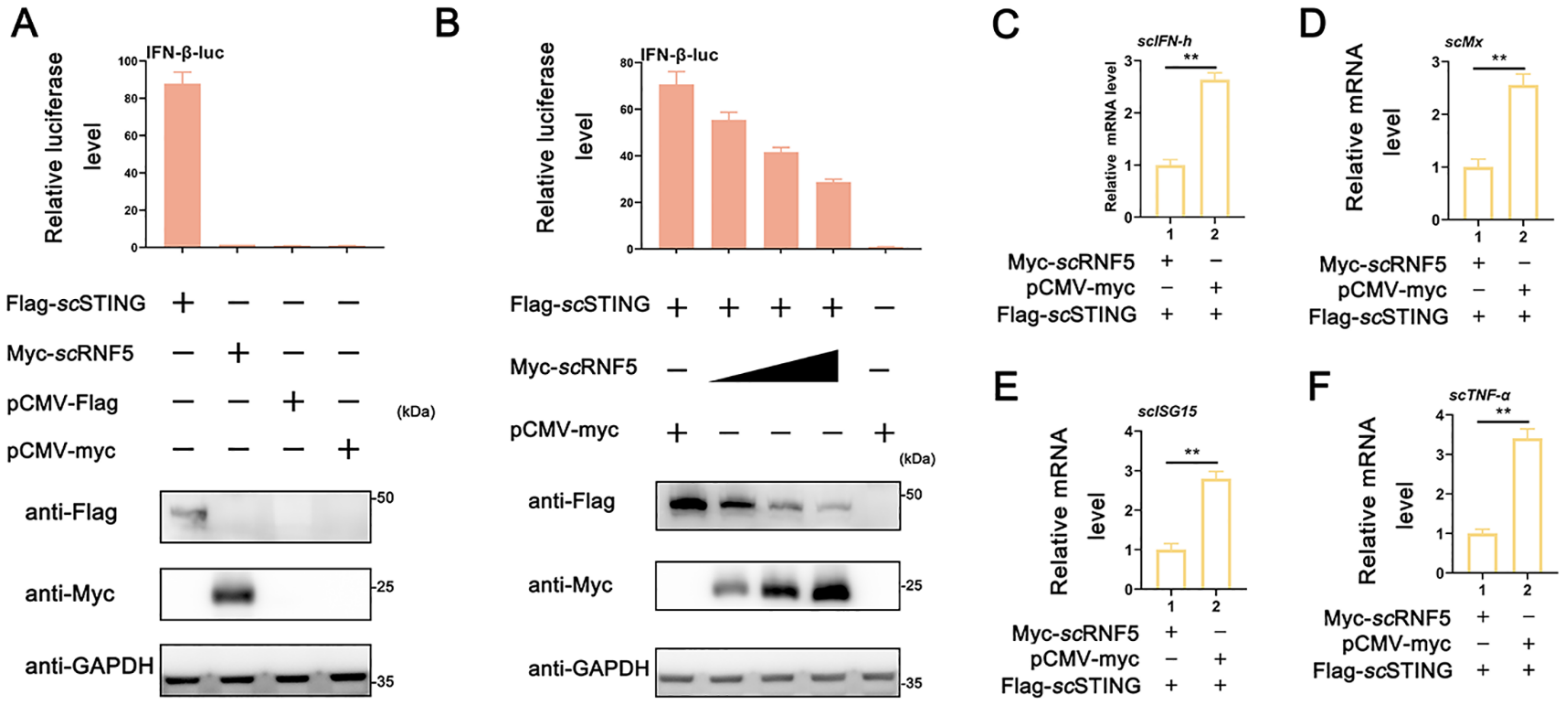
Suppression of *sc*STING-triggered IFN signaling by *sc*RNF5. **(A)** Relative luciferase activities of IFN-β-luc were measured relative to controls after cells were co-transfected with *sc*STING-flag and *sc*RNF5-myc. Cells transfected with pCMV-myc alone served as the negative control. **(B)** MFF-1 cells were co-transfected with the reporter plasmids pRL-TK and IFN-β-luc, as well as the indicated plasmids *sc*RNF5 and *sc*STING in 24-well plates. A reporter assay was performed 36 h post-transfection. **(C–F)** Cells were collected 24 h post-transfection after co-transfection *sc*RNF5 or vector alone alongside *sc*STING, and levels of expression levels of *scIFN-h* **(C)**, *scMx* **(D)**, *scISG15* **(E)** and *scTNF-α* **(F)** were assessed. Data shown are representative of three independent experiments (*n*=3). Statistical analysis revealed significant differences (**p < 0.01).

In mammals, STING serves as a key adaptor in innate immune responses triggered by pathogenic DNA or RNA, but its antiviral activity is attenuated by RNF5 (12). Previously, we found that overexpressing *sc*STING enhances IFN promoter activity in MFF-1 cells (25). To further explored the role of *sc*RNF5 in the IFN signaling pathway, MFF-1 cells were co-transfected with plasmids expressing *sc*RNF5 and/or *sc*STING, followed by a luciferase reporter assay. The results showed that *sc*STING, but not *sc*RNF5, significantly activated the IFN-β promoter. Notably, *sc*RNF5 inhibited *sc*STING-mediated IFN-β-luc activity in a dose-dependent manner when co-expressed with *sc*STING (**Figures 3A, B**). Furthermore, co-transfection experiments revealed that the expression levels of IFN-I signaling related genes (*scIFN-h*, *scMx*, *scISG15* genes; **Figures 3C–E**) and pro-inflammatory factor (*sc*TNF-α gene; **Figure 3F**) in cells co-expressing *sc*STING and *sc*RNF5 were significantly lower than those in cells co-expressing *sc*STING and control vector pCMV-myc (*p*<0.01). These results suggested that *sc*RNF5 might suppress the *sc*STING-mediated IFN signaling pathway.

### 3.5 *sc*RNF5 targeted *sc*STING for degradation via ubiquitination pathway

**Figure 4:**
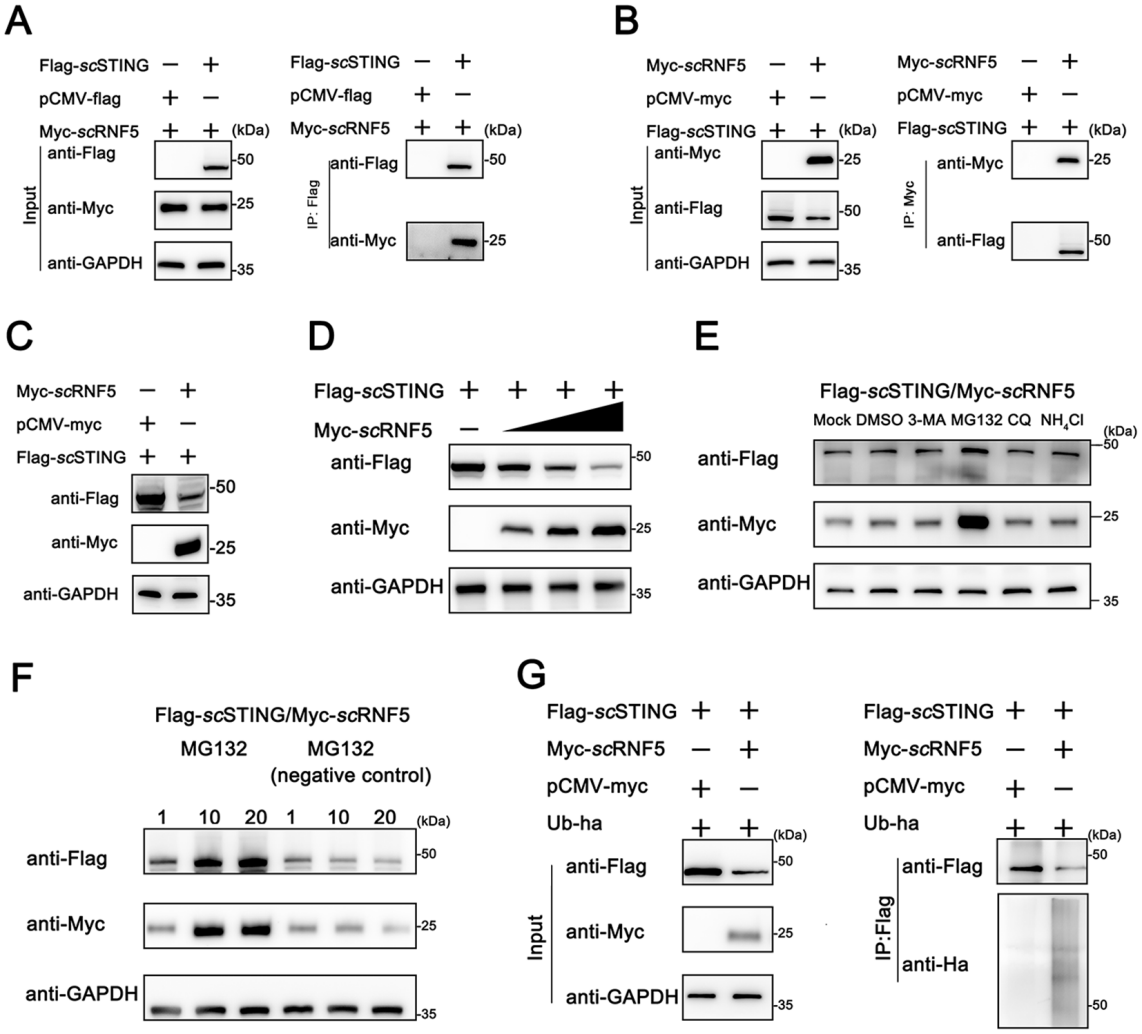
Ubiquitination-mediated degradation of *sc*STING by *sc*RNF5. **(A, B)** FHM cells were transfected with pCMV-Myc-*sc*RNF5 or empty vector and pCMV-Flag-*sc*STING plasmids. Co-IP and WB analyses were performed for detection. IB: immunoblotting; IP: immunoprecipitation. **(C)** Western blotting analyses were used for detection. FHM cells were transfected with pCMV-Myc-*sc*RNF5 or empty vector and pCMV-Flag-*sc*STING plasmids. **(D)** *sc*RNF5 overexpression leads to dose-dependent STING degradation. FHM cells were seeded into 6-well plates, incubated overnight, and co-transfected with 2 μg Flag-*sc*STING and Myc-*sc*RNF5 (0.5, 1, and 2 μg, respectively), After 24 h, lysates were immunoblotted with anti-Flag, anti-Myc, and anti-GAPDH antibodies. **(E)** The effects of MG132, 3-MA, CQ and NH_4_Cl on *sc*RNF5-induced *sc*STING degradation were assessed. FHM cells in 6-well plates were transfected with Flag-*sc*STING (2 μg) and Myc-*sc*RNF5 (2 μg). At 24 h post-transfection (hpt), cells were treated with DMSO or the respective inhibitors (MG132: 20 mM, 3-MA: 5 mM, CQ: 10 mM, NH_4_Cl: 20 mM). After 12 h, lysates were immunoblotted using anti-Flag, anti-Myc, and anti-GAPDH antibodies. **(F)** FHM cells were transfected and treated as in **(E)**, but with MG132 concentrations of 1, 10, and 20 mM, and a negative control (MG-132). Lysates were immunoblotted as before. **(G)** Ubiquitination of *sc*STING was assessed in FHM cells co-expressing Flag-*sc*STING, ha-ubiquitin, and either Myc-*sc*RNF5 (lane 2) or empty vector (lane 1). Flag-*sc*STING was immunoprecipitated with anti-Flag, and poly-ubiquitin chains were detected with anti-Ha.

To further explored the mechanism of how *sc*RNF5 regulated the *sc*STING IFN signaling pathway, we conducted a Co-IP assay to analyze the interaction between *sc*STING and *sc*RNF5. **Figure 4A** shows a distinct band representing *sc*RNF5 in the Flag-*sc*STING immunoprecipitated protein complex. Likewise, **Figure 4B** depicts a distinct band for *sc*STING in the Myc-*sc*RNF5 immunoprecipitated protein complex. These findings confirmed the interaction between *sc*RNF5 and *sc*STING. Upon co-transfection of *sc*RNF5 with *sc*STING, WB analysis showed a significant reduction in *sc*STING protein levels in the RNF5-expressing group (**Figure 4C**). To further investigate this effect, we employed a gradient of *sc*RNF5 expression and observed a clear inverse relationship; increasing *sc*RNF5 levels correlated with a progressive decrease in *sc*STING protein levels (**Figure 4D**). Next, we explored the mechanism by which *sc*RNF5 mediated *sc*STING degradation. Co-transfected of *sc*RNF5 and *sc*STING, followed by treatment with various drugs including MG132 (specific proteasome inhibitor), 3-MA (autophagy Inhibitor), CQ, and NH_4_Cl (lysosomal inhibitors), revealed that MG132 effectively blocked *sc*RNF5-mediated *sc*STING degradation (**Figure 4E**). Dose-dependent experiments with MG132 further confirmed that it inhibited *sc*RNF5-mediated *sc*STING degradation in a dose-dependent manner (**Figure 4F**). Based on these results, we hypothesized that *sc*RNF5 mediated *sc*STING degradation through ubiquitination. Ubiquitination experiments on *sc*STING in the presence of *sc*RNF5 revealed a significant increase in *sc*STING ubiquitination levels compared to control samples (**Figure 4G**). These observations suggested that *sc*RNF5 targeted *sc*STING for degradation via ubiquitination pathway.

### 3.6 *sc*RNF5 ubiquitination site on *sc*STING located in the first 180 aas

**Figure 5:**
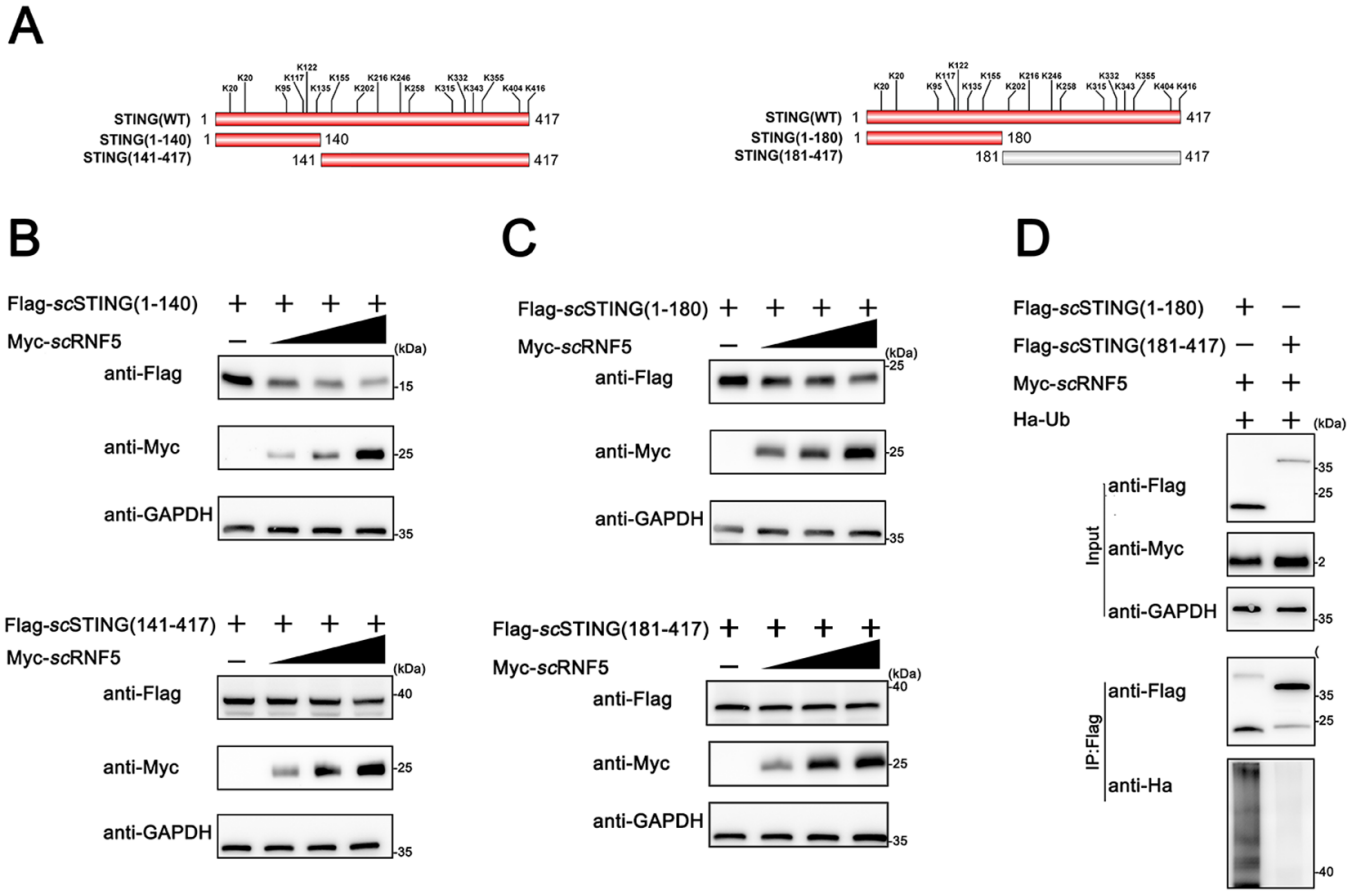
*sc*RNF5 ubiquitinated *sc*STING within its N-terminal 180 aas. **(A)** Lysine **(K)** residues positions in truncated STING variants. **(B)** FHM cells were seeded overnight in 6-well plates and co-transfected with 2 μg of either Flag-*sc*STING(1-140) or Flag-*sc*STING(141-417), along with Myc-*sc*RNF5 at concentrations of 0.5, 1, and 2 μg. After 24 h, lysates were immunoblotted using anti-Flag, anti-Myc, and anti-GAPDH antibodies. **(C)** Similarly, FHM cells were seeded and co-transfected as in **(B)**, but with Flag-*sc*STING(1-180) or Flag-*sc*STING(181-417). Lysates were processed for immunoblotting as before. **(D)** Ubiquitination of *sc*STING fragments was assessed. FHM cells were co-expressed with Myc-*sc*RNF5, Ha-ubiquitin, and either Flag-*sc*STING(1-180) or Flag-*sc*STING(181-417). Flag-scSTING was immunoprecipitated using anti-Flag, and poly-ubiquitin chains were detected with anti-Ha.

To identify the specific ubiquitination site of *sc*STING by *sc*RNF5, we constructed various truncation mutants of *sc*STING (**Figure 5A**) and conducted degradation experiments. Initial results showed that *sc*RNF5 degraded both *sc*STING (1–140) and *sc*STING(141-417) (**Figure 5B**). However, further segmentation analysis showed that *sc*RNF5 only degraded *sc*STING(1-180), but not *sc*STING (181-417) (**Figure 5C**), implying the ubiquitination site is located within the first 180 aas of *sc*STING. Moreover, ubiquitination experiments on *sc*STING(1-180) and *sc*STING(181-417) confirmed significant ubiquitination of *sc*STING(1-180) by *sc*RNF5 (**Figure 5D**), further indicating that the ubiquitination occurred within the 1 to 180 aas region.

### 3.7 *sc*RNF5 catalyzed the ubiquitination of *sc*STING at two lysine residues K135 and K155

**Figure 6:**
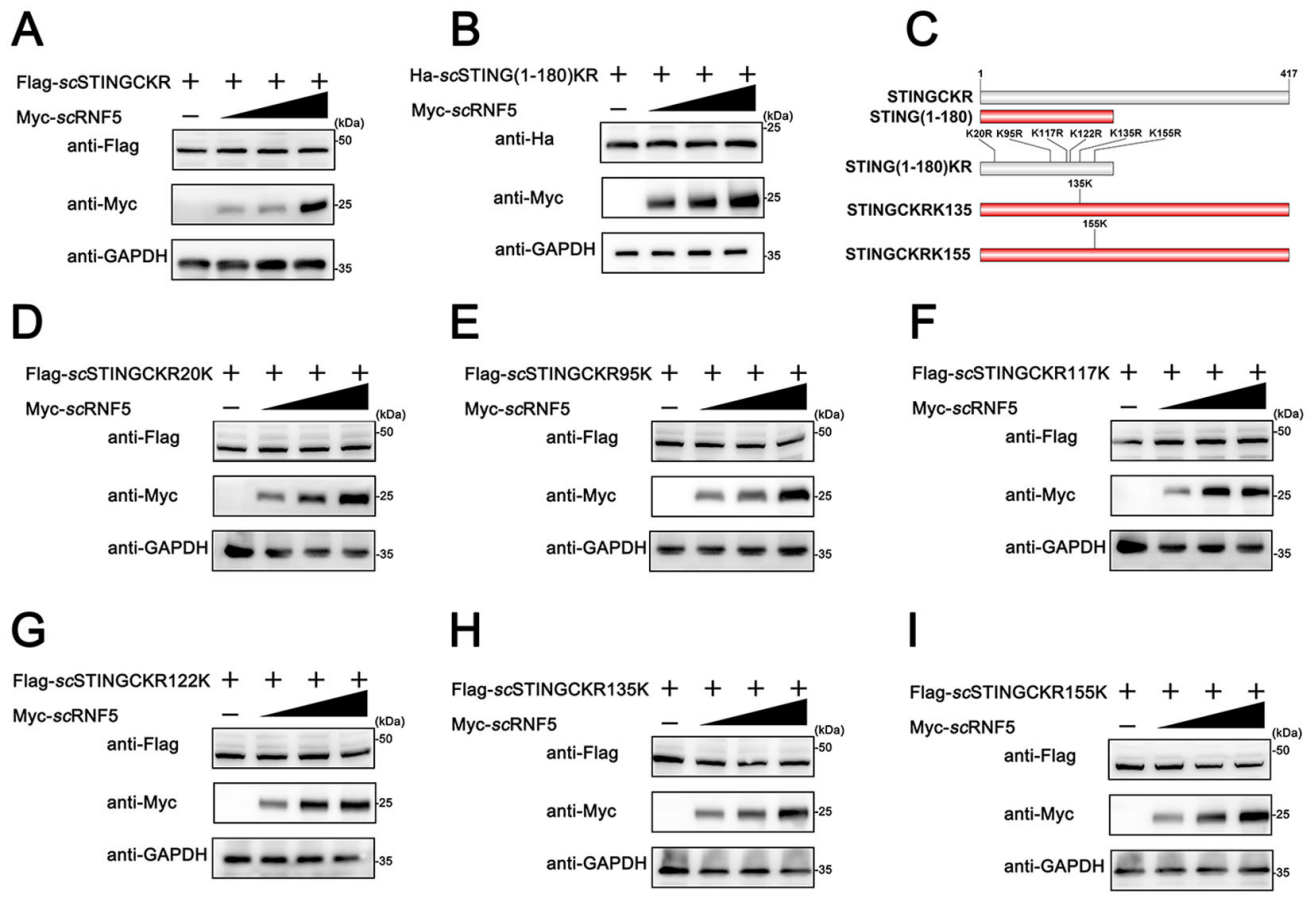
*sc*RNF5 targeted *sc*STING for ubiquitination at K135 and K155. A **(A, B)** FHM cells were co-transfected with 2 μg of Flag-*sc*STINGCKR/Ha-*sc*STING(1-180)KR and Myc-*sc*RNF5 (0.5, 1 and 2 μg) for 24 h. Lysates were then IB with anti-Ha, anti-Myc, and anti-GAPDH Abs. **(C)** Schematic of *sc*STING mutants used in this study. **(D–I)** FHM cells were co-transfected with 2 μg of various Flag-tagged *sc*STING mutants (Flag-*sc*STINGKR20K, Flag-*sc*STINGKR95K, Flag-*sc*STINGKR117K, Flag-*sc*STINGKR122K, Flag-*sc*STINGKR135K or Flag-*sc*STINGKR155K) and Myc-*sc*RNF5 (0.5, 1 and 2 μg) for 24 h. Lysates were then subjected to immunoblotted with anti-Flag, anti-Myc, and anti-GAPDH Abs.

Ubiquitination primarily targets lysine residues in various proteins, although other aas, like cysteine, can also undergo this post-translational modification (25). Mammalian RNF5 suppresses K48-linked ubiquitination at the K150 site, promoting proteasomal degradation (12). To investigated RNF5-mediated ubiquitination of STING involves amino acid residues in mandarin fish, we substituted all C and K residues in *sc*STING with R, generating a variant named *sc*STINGCKR. Moreover, we constructed a variant where all K residues in *sc*STING(1-180) were replaced with R, named *sc*STING(1-180)KR. Degradation experiments observed that *sc*RNF5 could no longer degrade *sc*STINGCKR (**Figure 6A**) and *sc*STING(1-180)KR (**Figure 6B**), suggesting *sc*RNF5-mediated ubiquitination of *sc*STING specifically targeted lysine residues. As shown in **Figure 6C**, the 1-180 aa region of *sc*STING contains six lysine residues. Next, we generated six mutants, each restoring one of the original K residue in the *sc*STINGCKR, and designed *sc*STINGCKR20K, *sc*STINGCKR95K, *sc*STINGCKR117K, *sc*STINGCKR122K, *sc*STINGCKR135K, and *sc*STINGCKR155K, respectively. We then performed co-transfection with the mutants and *sc*RNF5 was to identify the specific K residue targeted for degradation. The results showed that *sc*RNF5 had no degradation effect on *sc*STINGCKR20K, *sc*STINGCKR95K, *sc*STINGCKR117K, *sc*STINGCKR122K (**Figures 6D–G**). However, *sc*RNF5 regained its ability to degrade *sc*STINGCKR when K mutations were restored at positions K135 and K155 (**Figures 6H–I**). Those results suggested that *sc*RNF5 catalyzed the ubiquitination of *sc*STING specifically at lysine residues K135 and K155.

**Figure 7:**
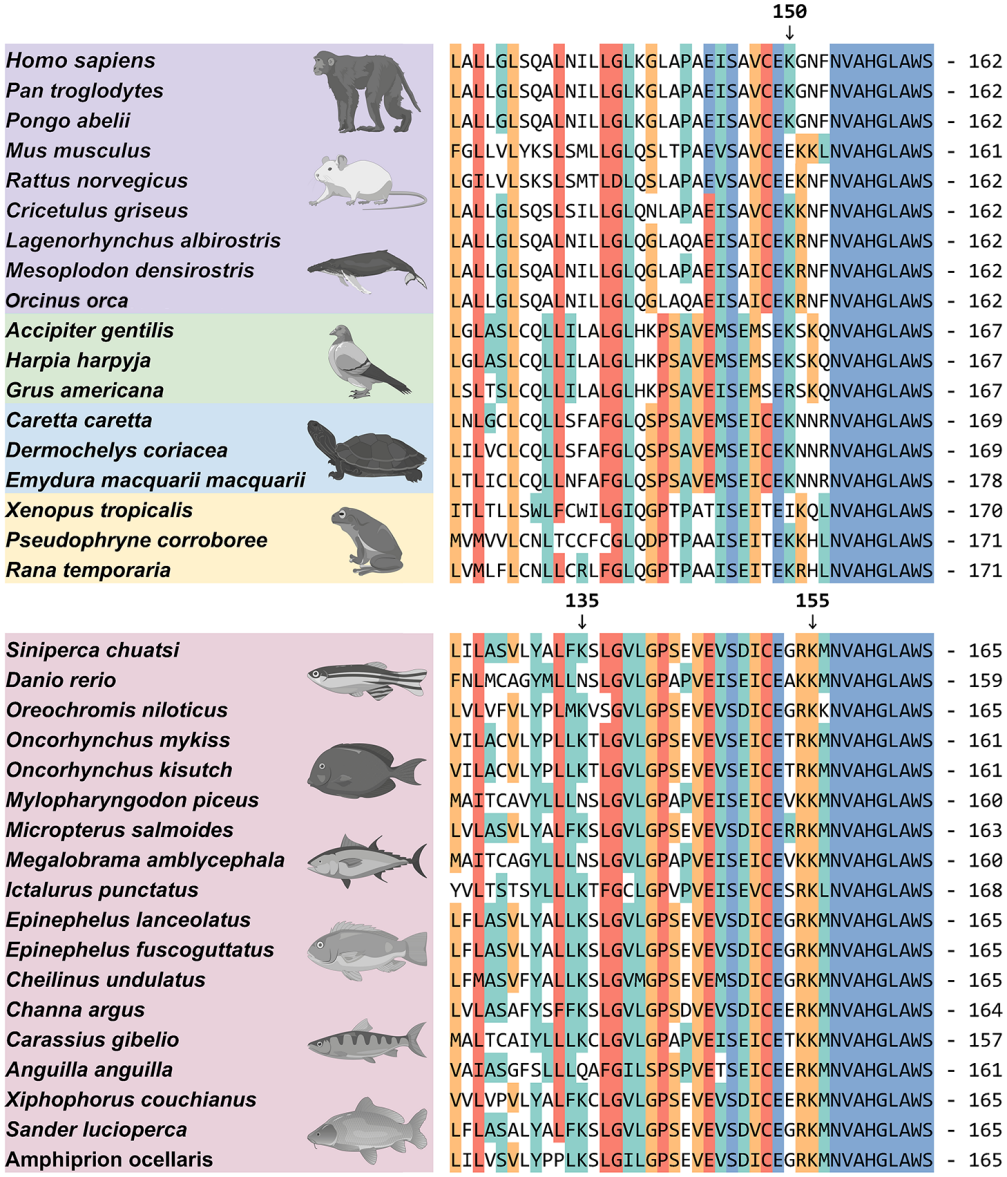
Protein sequence alignment of STING. The protein amino acid alignment reveals the conserved aa sequences and lysine site distributions of STING across different species. Accession numbers for these sequences were provided in the **Supplementary Material 2**. Arrows indicate lysine at the 150th position in *hs*STING and lysine at the 135th and 155th positions in *sc*STING.

Furthermore, to compare the differences in *sc*RNF5 targeting of *sc*STING lysine residues between mammals and other vertebrates, we performed homologous sequence alignment of STING proteins from mammals, birds, reptiles, amphibians and fish. Interestingly, STING from most fish species contains two lysine residues at positions K135 and K155, whereas STING from other vertebrates (mammals, birds, reptiles, and amphibians) does not contain this two lysine (**Figure 7**), suggesting potential differences in the negative regulation of STING in teleost fish compared to other vertebrates.

### 3.8 Negative regulation of *sc*STING-mediated antiviral activity by *sc*RNF5

**Figure 8:**
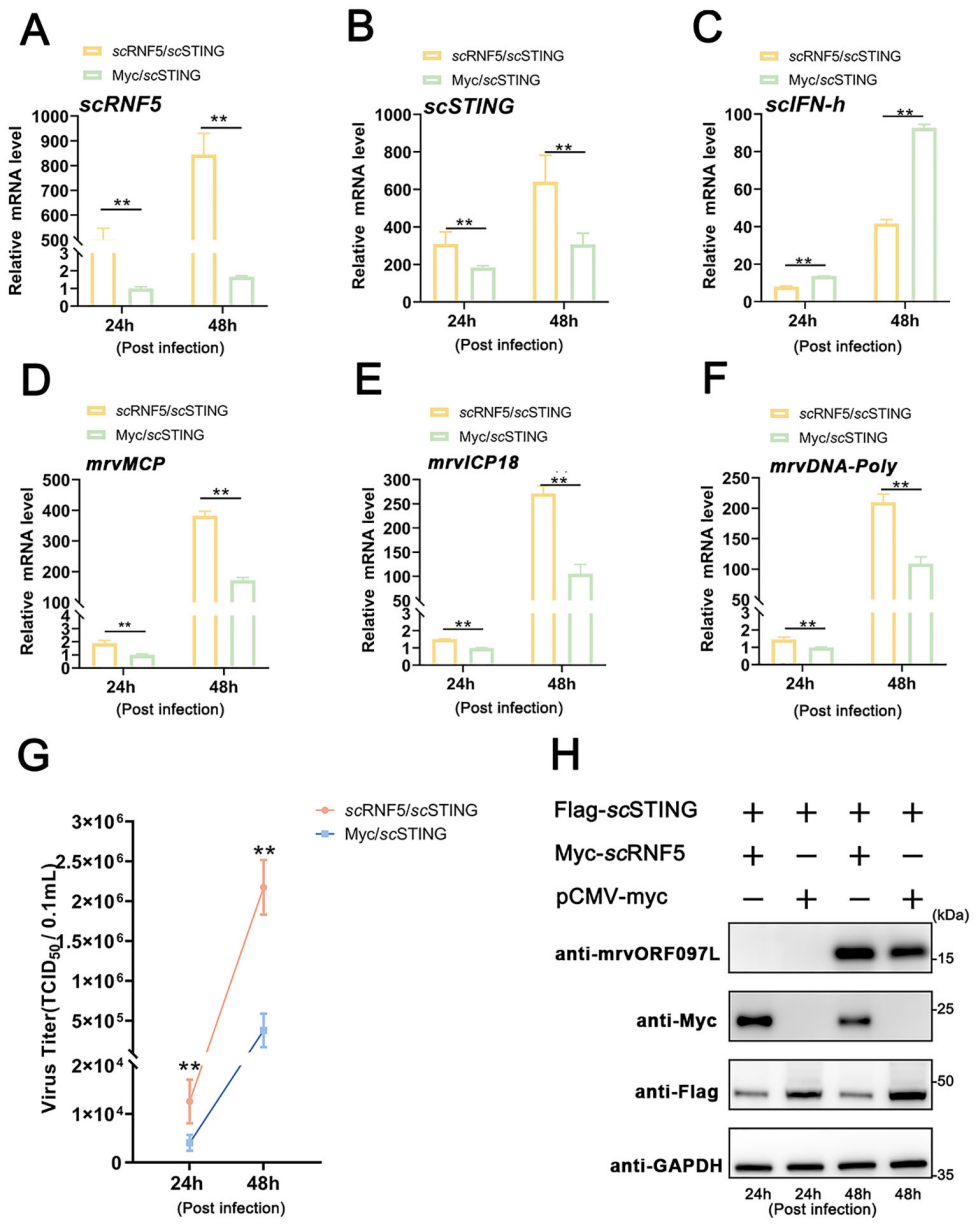
*sc*RNF5 inhibited *sc*STING-mediated antiviral activity. **(A)** MFF-1 cells were infected with MRV and harvested for RT-qPCR, WB, and virus titers analysis independently at the indicated time points. Expression levels of *sc*RNF5 **(A)**, *sc*STING **(B)**, *scIFN-h* **(C)**, *mrvMCP* **(D)**, *mrvICP18* **(E)**, *mrvDNA-Poly* **(F)** in MRV-infected cells at indicated times. For qRT-PCR analysis, the baseline was established as the group exhibiting the lowest relative expression among all groups, with this value set to 1. All groups represented different time points following viral infection. **(G)** Virus titers were measured on a 96-well cell culture plate using the finite dilution method. **(H)** WB analysis was performed to detect levels of viral protein mrvORF097L. The left two lanes indicate samples taken at 24 h post-infection, while the right two lanes indicate samples taken at 48 h post-infection. Vertical bars represent ± SD (*n* = 3). Statistical significance is indicated by asterisks, with ** referring to *p* < 0.01.

To investigate whether the antiviral ability of *sc*STING was also regulated by *sc*RNF5, MFF-1 cells co-expressing *sc*RNF5 and *sc*STING were infected with MRV. Viral titer was then assayed from collected supernatants, while cells were harvested for qRT-PCR and WB analysis. The results showed that cells co-expressing *sc*STING and *sc*RNF5 (**Figures 8A, B**) had increased the expression levels of the *scIFN-h* gene (**Figure 8C**) and MRV viral genes (*mrvMCP*, *mrvICP18*, *mrvDNA-Poly*) (**Figures 8D–F**). In addition, these cells exhibited higher viral titers (TCID_50_) and more abundant viral proteins (mrvORF097L) compared to control cells transfected with *sc*STING alone (**Figures 8G, H**). These results suggested that the significant negative regulation of *sc*RNF5 on scSTING-mediated antiviral innate immunity.

## 4 Discussion

IFNs are crucial antiviral agents in innate immunity, but their unregulated production can harm the organism, potentially leading to severe outcomes. Therefore, maintaining balanced IFN production is essential for organism homeostasis (43). Upon activation, STING undergoes post-translational modifications, such as phosphorylation and ubiquitination (44). TRIM56 induce K63-linked ubiquitination at residue K150 of STING, thereby activating the STING/IFN signaling pathway (45). Autocrine motility factor receptor facilitates STING activation through K27-linked ubiquitination at multiple sites (46). Conversely, human ubiquitin specific peptidase 13 suppresses K27-linked ubiquitination of STING, reducing its interaction with TBK1 (47). Our study showed that RNF5 regulated STING by facilitating its ubiquitination and destabilization, thereby negatively modulating the activity of STING in mandarin fish.

One key ubiquitination and regulation site on mammalian STING is K150. RNF5 suppresses K48-linked ubiquitination at the K150 site, promoting proteasomal degradation (12). RNF26 enhances STING stability by facilitating K11-linked polyubiquitination at K150, while inhibiting RNF5-induced STING ubiquitination (48). The deubiquitinase cylindromatosis protein removes K48-linked ubiquitination at K150 (49). Our data showed that *sc*RNF5-mediated ubiquitination of *sc*STING at two lysine residue leads to proteasome degradation. *sc*RNF5 regulated two ubiquitination sites on *sc*STING: K155 (corresponding to mammalian STING K150) and an extra site at K135. Ubiquitination at either of these sites is sufficient to trigger degradation, suggesting potential differences in the negative regulation of STING between teleost fish and other vertebrates.

Furthermore, E3 ligase plays a pivotal role in substrate determination during ubiquitination and have been extensively studied. Recent research has shown that in black carp, RNF5 regulates STING degradation through the K48-linked ubiquitin-proteasome pathway (27). Black carp RNF5 also interacts with MAVS, negatively regulating the MAVS/IFN signaling pathway (26). Blood plays a vital role in the immune system, particularly in mounting immune responses. The tissue distribution and expression of RNF5 in mandarin fish during MRV infection reveal a high level of expression in the blood, suggesting RNF5 might contribute to the immune response in mandarin fish. Furthermore, in terms of virus promotion, our data showed that *sc*RNF5 overexpression in MFF-1 cells significantly suppressed IFN/ISG transcription, augmented MRV-encoded proteins, and increased virus titers, attenuating STING-mediated IFN expression and antiviral activity.

Our findings suggest several promising strategies for preventing and controlling MRV disease in mandarin fish. One approach involves incorporating *sc*RNF5 inhibitors into mandarin fish feed, which may modulate immune responses and reduce MRV infection; nevertheless, additional research is needed to confirm its efficacy and safety. Another strategy entails identifying the K135/155 mutant scSTING in wild mandarin fish, providing a valuable genetic resource for breeding disease-resistant strains. Once these mutants are identified, they can be integrated into selective breeding programs to enhance the disease resistance of farmed fish. Furthermore, introduction of R135/155 *sc*STING into mandarin fish via transgenic techniques offers an innovative approach to bolster their antiviral defenses. Although this strategy is promising, it also poses challenges in technology, regulation, and public acceptance. Overall, these strategies have the potential to substantially improve both the disease resistance and production efficiency of mandarin fish aquaculture.

**Figure 9:**
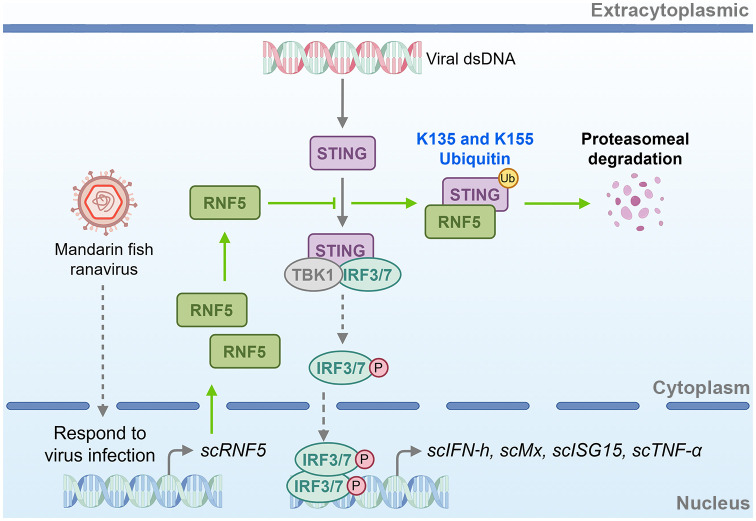
Schematic of RNF5-mediated negative regulation of STING signaling in mandarin fish. *sc*RNF5 interacts with *sc*STING, ubiquitinating lysine residues at positions 135 and 155. This ubiquitination marks *sc*STING for proteasomal degradation, preventing IFN antiviral interferon response. By promoting *sc*STING degradation, *sc*RNF5 suppresses the host’s antiviral immune response, thereby facilitating viral replication.

In summary, our study highlighted that RNF5 negatively regulated the STING-mediated IFN signaling pathway in mandarin fish, attenuated STING’s antiviral capacity, and facilitated STING degradation via the ubiquitin-proteasome pathway at K135 and K155 resides (**Figure 9**). This study offers valuable insights into the regulatory mechanisms of STING-mediated signaling in teleost fish, presenting novel prospects for disease-resistant breeding and drug target identification, thereby paving the way for further elucidation.

## Data Availability

The original contributions presented in the study are included in the article/**Supplementary Material**. Further inquiries can be directed to the corresponding author.

## Supplementary material

Supplementary Material 1 Primers used in this study.

Supplementary Material 2 Accession numbers of RNF and STING family members sequences in the NCBI.
